# Clinical Efficacy and Safety of Silicone Elastomer Sheet during Decompressive Craniectomy: Anti-Adhesive Role in Cranioplasty

**DOI:** 10.3390/brainsci11010124

**Published:** 2021-01-18

**Authors:** Young Ha Kim, Chi Hyung Lee, Chang Hyeun Kim, Dong Wuk Son, Sang Weon Lee, Geun Sung Song, Soon Ki Sung

**Affiliations:** 1 Department of Neurosurgery, Pusan National University Yangsan Hospital, Yangsan 50612, Korea; twins728@hanmail.net (Y.H.K.); gto270@naver.com (C.H.L.); mole83@hanmail.net (C.H.K.); md6576@naver.com (D.W.S.); sangweonlee@pusan.ac.kr (S.W.L.); gnsong@pusan.ac.kr (G.S.S.); 2 Research Institute for Convergence of Biomedical Science and Technology, Pusan National University Yangsan Hospital, Yangsan 50612, Korea; 3 Department of Neurosurgery, Pusan National University School of Medicine, Yangsan 50612, Korea

**Keywords:** decompressive craniectomy, cranioplasty, silicone elastomers, tissue adhesions

## Abstract

(1) Background: Cranioplasty is a surgery to repair a skull bone defect after decompressive craniectomy (DC). If the process of dissection of the epidural adhesion tissue is not performed properly, it can cause many complications. We reviewed the effect of a silicone elastomer sheet designed to prevent adhesion. (2) Methods: We retrospectively reviewed 81 consecutive patients who underwent DC and subsequent cranioplasty at our institution between January 2015 and December 2019. We then divided the patients into two groups, one not using the silicone elastomer sheet (*n* = 50) and the other using the silicone elastomer sheet (*n* = 31), and compared the surgical outcomes. (3) Results: We found that the use of the sheet shortened the operation time by 24% and reduced the estimated blood loss (EBL) by 43% compared to the control group. Moreover, the complication rate of epidural fluid collection (EFC) in the group using the sheet was 16.7%, which was lower than that in the control group (41.7%, *p* < 0.023). Multivariate logistic regression analysis showed the sheet (OR 0.294, 95% CI 0.093–0.934, *p* = 0.039) to be significantly related to EFC. (4) Conclusions: The technique using the silicone elastomer sheet allows surgeons to easily dissect the surgical plane during cranioplasty, which shortens the operation time, reduces EBL, and minimizes complications of EFC.

## 1. Introduction

Decompressive craniectomy (DC) is an effective surgical method to control increased intracranial pressure, swelling, and increased cerebral blood flow in acute brain injury, such as intracranial hemorrhage (ICH), traumatic brain injury, and cerebral infarction [1,2,3,4]. In patients who survive DC, cranioplasty (CP) is required at the time when the cerebral edema has subsided and patient condition is stable. The primary aim of CP is to protect the brain, which, without a bony cover, is prone to injury. CP procedure is also performed for cosmetic purposes [5,6]. Moreover, several studies have reported that CP improves neurological function and affects cerebral glucose metabolism, postural blood flow regulation, and cerebrospinal fluid (CSF) flow [7,8,9].

Complications that may occur after CP include bone flap absorption, infection, and postoperative hemorrhage [10,11,12]. Particularly, during the process of dissection between the musculocutaneous flap layer and the dura layer, if adhesions are severe and the dissection is insufficient, then the operation time is prolonged. If the layer is not properly located, then the injury to the brain surface and temporalis muscle may result in brain cortical hemorrhage, CSF leakage, and epidural hemorrhage [13,14,15]. To reduce such complications, several studies have devised a method to make dissection easier by placing various anti-adhesive substances such as expanded polytetrafluoroethylene membranes, gelatin films, and bovine pericardial patches between the dura mater and musculocutaneous flaps to prevent adhesion. It has been reported that operation time and blood loss can be reduced via this new method [13,14,15,16,17,18,19,20]. However, many studies have been limited only to trauma patients or patients with spontaneous cerebral hemorrhage, and there have been few comparative and control studies between groups with and without anti-adhesion material. In addition, the clinical outcome of the patient has not been studied in detail. Therefore, further research is needed.

In our center, a collagen matrix dural substitute matrix “DuraGen^®^” (Integra Life Science Inc, Plainsboro, NJ, USA) is placed onlay or inlay of the native dural leaflets for duraplasty, and a silicone elastomer sheet (Medical Grade Silicone Sheeting, BioPlexus Corporation, Los Angeles, LA, USA) is used as a cover to prevent adhesion between the dura and musculocutaneous flap. To date, few studies have been conducted using silicone elastomer sheets. Therefore, we performed a comparative control study to evaluate the clinical outcomes of its use.

## 2. Materials and Methods

### 2.1. Patient Population

We retrospectively reviewed the medical records of 95 patients who underwent DC with subsequent CP at our institution between January 2015 and December 2019. DC was performed in cases of traumatic brain injury, such as ICH, subdural hemorrhage (SDH), epidural hemorrhage (EDH), and fracture compound comminuted depressed (FCCD) of the skull. DC was also performed in cases of nontraumatic brain injury, such as spontaneous ICH, subarachnoid hemorrhage from ruptured cerebral aneurysm, and brain edema after cerebral infarction. These patients were divided into group A, in which the silicone elastomer sheet was not used, and group B, in which the silicone elastomer sheet was used. Within the group, subgroups were divided into early and late CP based on 90 days. CP variables were reduced by including only the surgeries performed by 2 skilled surgeons. CP also included surgery that used only an autologous bone flap. The exclusion criteria were as follows: (1) Pediatric patients under 19 years of age, (2) patients who had cerebellar surgery, (3) patients who underwent ventriculoperitoneal (VP) shunt at the same time as CP, and (4) patients who underwent bilateral craniectomy and CP on both sides. The study protocol was approved by the institutional review board (IRB) and medical ethics committee.

### 2.2. Operative Techniques

#### 2.2.1. DC Technique

**Figure 1:**
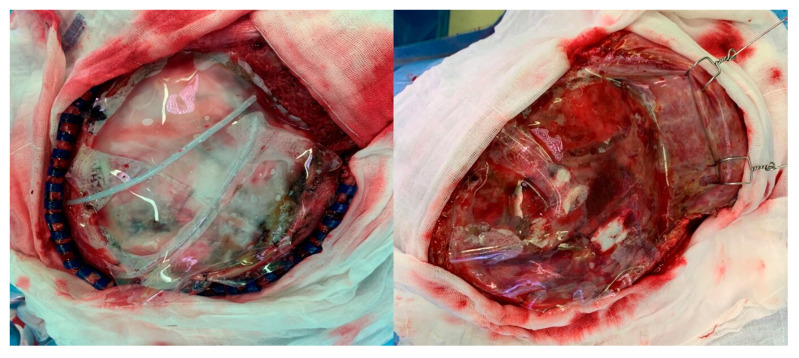
Intraoperative photos. (**A**): After duraplasty, the silicone elastomer sheet cut according to the size of the bone defect was placed on the epidural space and loosely sutured to the bone margin and fixed during decompressive craniectomy. (**B**): Epidural space after dissection on the sheet during cranioplasty.

All patients underwent fronto-temporo-parietal hemicraniectomy. The surgical procedure started with a large, unilateral, curvilinear incision. The incision began anterior to the tragus and below the upper edge of zygomatic arch and followed a “question mark” shape, curving above the upper edge of the pinna, then turning just above the transverse sinus to the external occipital protuberance, and then extending cranially to the vertex, anteriorly to behind the hairline and away from midline. A skin flap was peeled off with the temporalis muscle from the periosteum, and a myocutaneous flap was reflected frontally and hooked. Multiple burr holes were made on the exposed skull bone, and craniotomy was performed to remove the fronto-temporo-parietal bone flap [21]. The additional temporal bone was removed from the squamous part down to the temporal floor. The dura was opened with a stellate-shaped incision placed 1 cm away from the bone edge, and the collagen matrix DuraGen^®^ was used to cover the exposed brain cortex. Dural leaflets were sutured loosely and DuraGen^®^ was added to prevent CSF leakage. In group B, a 152 mm × 203 mm × 0.025 mm silicone elastomer sheet was placed between the dura and the myocutaneous flap to prevent adhesion. The silicone elastomer sheet was cut to sufficiently cover the bone defects, and a suture was performed on the bone edge loosely to avoid interfering with the recovery of the midline shift. Two drains were placed on the top and bottom of the silicone elastomer sheet (Figure 1A) The muscle and skin flap were sutured layer by layer and the procedure was completed. The excised bone flap was stored in the bone bank at −80 °C in the hospital immediately after DC.

#### 2.2.2. CP Technique

CP was performed between 2 weeks and 8 months after DC, and an autologous bone flap was used in all cases. In patients with reduced brain swelling while admitted to the Department of Rehabilitation after decompressive surgery, cranioplasty was performed early before discharge. In the case of patients who were discharged from the hospital and underwent follow-up in the outpatient clinic, cranioplasty was performed with elective operation in a state where surgery was possible depending on the anesthesia evaluation and the patient’s general condition.

The epidural space was opened by performing an incision along the previous surgical incision line and dissection between the dura and the myocutaneous flap. In group B, the sheet was found after skin incision, and dissection proceeded along this sheet (Figure 1B). The sheet was removed and checked for a dura defect or CSF leakage. For areas with dura defects, duraplasty was performed using DuraGen^®^. The preserved bone flap was removed and fixed using plates and screws. A drain was placed on the skull bone, the myocutaneous flap was sutured layer by layer, and the surgery was completed.

### 2.3. Clinical Outcomes Evaluation

We reviewed parameters including history, age, sex, underlying disease, anticoagulant/antiplatelet agent medication, laboratory test results, time interval between DC and CP (days), length of hospital stay after cranioplasty, follow-up periods, and postoperative complications from their medical records. The complications after CP were postoperative infection, hydrocephalus, postoperative hemorrhage, and epidural fluid collection. Postoperative infection included intracranial and superficial surgical site infections (SSIs). Dura tear and duraplasty were referred to in the surgical records. Furthermore, we reviewed preoperative and postoperative brain computed tomography (CT) scans and confirmed the diagnosis, postoperative bleeding or fluid collection, and midline shift differences. Epidural fluid collection was defined as CT showing low-density fluid over 5 mm in the epidural space within 7 days after CP. Anesthesia records were reviewed to check the estimated blood loss (EBL) and operation time. EBL was measured as the difference between the amount of blood on the gauge or drap during surgery, the amount of irrigation used during surgery, and the amount of suction measured after surgery. Although accurate measurements are difficult, a relative comparison can be made since all surgeries were measured in the same way as described above.

### 2.4. Statistical Analysis

Continuous variables were expressed as mean ± standard deviation, and categorical variables were expressed as frequency and percentage (%). Group differences in clinical outcomes were evaluated using Student t-tests and Mann–Whitney U tests for continuous variables and chi-square test and Fisher’s exact test for categorical variables. Adjusted odds ratios and 95% confidence intervals (CIs) were reported for the factors included in the multivariate logistic regression model. All analyses were performed using SPSS Statistics version 26.0 (IBM Co., Armonk, NY, USA). Statistical significance was set at *p* < 0.05.

## 3. Results

### 3.1. Baseline Characteristics

After excluding 14 patients who did not meet the set criteria, 81 patients were enrolled in the study. Of the 81 patients enrolled, 51 patients were in group A (without silicone elastomer sheet) and 30 patients were in group B (with silicone elastomer sheet). The mean age in group A was 56.3 ± 13.1 years and that in group B was 57.0 ± 13.1 years. There was no significant difference in baseline characteristics between the two groups (Table 1).

### 3.2. Difference between Complications and Midline Shift between the Two Groups after DC

After DC, complications were investigated to determine if the silicone elastomer sheet influenced infection, bleeding, and brain decompression. Postoperative hemorrhage occurred in five patients in group A, four of whom underwent repeat surgery. This complication was also observed in four patients in group B, of whom one underwent repeat surgery. However, there was no statistically significant difference (*p* = 0.551) between the two groups. The midline shift difference between the brain CT taken a week after DC and the CT taken immediately after surgery was investigated to compare and analyze whether the silicone elastomer sheet inhibited brain decompression. The mean values were 3.8 mm and 3.2 mm in groups A and B, respectively, with no statistically significant difference (*p* = 0.423). These results show that even if the sheet forms a barrier, it does not affect the recovery of midline shift (MLS) (3.8 vs. 3.2, *p* = 0.423). It is also important to note that no postoperative infection was observed in either group (Table 2).

### 3.3. CP Results in the Two Groups

The mean time interval between DC and CP was 85.0 ± 48.4 days for group A and 72.8 ± 40.8 days in group B, but there was no statistically significant difference (*p* = 0.251). Group B showed statistically significantly shorter operation time (129.1 ± 35.9 vs. 98.3 ± 37.8, *p* < 0.001) and less EBL than group A (235.2 ± 189.8 vs. 133.3 ± 99.4, *p* < 0.002). Thus, using a silicone elastomer sheet, the operation time was shortened, and the EBL was reduced. Moreover, group B had a statistically significantly lower complication rate of epidural fluid collection (EFC) (41.7% vs. 16.7%, *p* = 0.023). The mean EFC depth was 8.71 mm in group A and was slightly thicker in group B at 9.63 mm. There were four cases in group A with MLS, which is more than in group B. However, none of these patients underwent repeat surgery. There was one postoperative infection in each group, and both received repeat surgery. In group A, postoperative brain cortical injuries occurred in four patients, more than in group B. In group A, hydrocephalus also occurred in two patients, and VP shunt surgery was performed. However, there was no statistically significant difference between the two groups. The mean hospital admission period was 13.0 ± 3.6 days in group A and 12.4 ± 4.3 days in group B, with no significant difference between the two groups. The mean follow-up period was 75.6 ± 72.2 days for group A and 57.8 ± 72.6 days for group B. Group A was followed up for a longer period of time, but there was no statistical difference (Table 3).

### 3.4. Risk Factor Analysis of Epidural Fluid Collection after Cranioplasty

Among complications, the relationship between EFC and each factor that showed a significant difference was analyzed. Factors affecting EFC included the silicone elastomer sheet, epidural air bubble, dural calcification, and late CP timing (Table 4). On multivariate logistic regression analysis, the silicone elastomer sheet (OR 0.294, 95% CI 0.093–0.934, *p* = 0.039) and epidural air bubble (OR 3.809, 95% CI 1.383–10.490, *p* = 0.010) were found to be independent risk factors for EFC (Table 5).

### 3.5. Comparison of Results in Trauma and Non-Trauma in Group B

In order to confirm that there was no difference between trauma patients and non-trauma patients when silicone elastomer sheets were used, the relationship between each factor was analyzed after subgrouping in group B. As a result, there was no statistical difference between the two groups in terms of EBL, operation time, EFC, infection, MLS difference after DC (Table 6).

## 4. Discussion

### 4.1. Advantage of Anti-Adhesive Materials

If the surgical plane cannot be found between the dura and the musculocutaneous flap during CP, complications such as postoperative bleeding, muscle injury, and CSF leakage may occur [13,14,15,22]. Accordingly, various anti-adhesive materials have been reported to prevent epidural adhesion and help to easily detect the surgical plane. To date, the use of synthetic or biological patches such as expanded polytetrafluoroethylene membrane (ePTFE) [14,17,20], gelatin film [23], OrthoWrap™ (bio-absorbable sheet; MAST Biosurgery, San Diego, CA 92121, USA) [24], sodium hyaluronate plus carboxymethylcellulose [25], bovine pericardial patch [13,26,27], and epidural multi-slitted microporous polyesterurethane [28] have been reported, and their use has facilitated dissection, reducing operation time and EBL. A silicone elastomer sheet was used in our institution, and a comparative control study was conducted.

In our study, the advantage of using the silicone elastomer sheet was that the operation time was shortened by approximately 24% and EBL was reduced by 43% compared to the control group. There was no adhesion between the dura and the musculocutaneous flaps above and below the silicone elastomer sheet. Once the silicone elastomer sheet was found in the bone margin, the surgeon could easily perform dissection, thereby reducing the operation time. In addition, as the dissection became easier, there were fewer injuries of the myocutaneous tissue, so EBL was also low. Other comparative control studies using anti-adhesion materials have shown similar results [16,17,24]. Vakis et al. reported that the use of ePTFE shortened the operation time by approximately 25% and reduced EBL by 37% compared with the control group [17]. As in our case, Lee et al., who used a silicone elastomer sheet, showed that the operation time and EBL showed statistically significant reduction [16]. Moreover, in a comparative study by Khalili et al. using a bio-absorbable sheet (OrthoWrap™) for patients with traumatic brain injury, the operation time was shorter and EBL was less [24].

In the present study, there was no statistical difference in the infection rate between the two groups, but it has been reported that prolonged operation time increases the incidence of SSI. Shibahashi et al. reported that there was a significant relationship between operative time and SSI, and the relative risk ratio was 7.4 in patients with an operative time of >98 min [29]. In addition, it has been reported that the infection rate decreases when the temporalis muscle has little injury, and the use of a sheet can further reduce the occurrence of infection [30]. In CP, EBL has never been suggested as a risk factor, but if intraoperative blood loss increases, transfusion may be necessary. It has been reported that transfusion increases complications in other surgical fields. Thus, it is important to reduce EBL [31,32].

### 4.2. Safety of Silicone Elastomer Sheet

The silicone elastomer sheet is a flexible and transparent silicone elastomer material designed for various medical and laboratory applications. The silicone elastomer sheet is widely used in general surgery, plastic surgery, and otolaryngology surgery because it can prevent adhesion to surrounding tissue and form a barrier [33,34,35]. Complications of inflammation, infection, migration, and foreign material reaction have been reported in craniofacial surgery using a thicker sheet than ours, but these complications were reported in patients who had received a permanent insertion for a very long time [33]. Therefore, the comparison of the incidence of these complications in CP, in which the sheet is removed within approximately 1 year, is not appropriate. There is currently only one report using a silicone elastomer sheet for CP, in which postoperative infection was reported in one patient (4%, *n* = 1/24) after CP but no other complications were described [16]. In our study, there was no infection in patients with the silicone elastomer sheet before CP, and no postoperative infection occurred in patients who had CP at the last 8 months. Furthermore, colonization, inflammation, and infection did not occur in one patient with brain abscess using the silicone elastomer sheet. The migration of the silicone elastomer sheet can also be problematic, but this problem was solved by adding four to five sutures to the bone margin. Moreover, it can be thought that, because the silicone elastomer sheet forms a barrier, it can affect brain decompression. However, our results show that the silicone elastomer sheet does not affect MLS recovery (3.8 vs. 3.2, *p* > 0.423). Given these features, the silicone elastomer sheet can be used safely in DCs.

### 4.3. Efficacy of Silicone Elastomer Sheet

Although EFC is not a major complication such as bone flap resorption and infection, it is a complication that may require reoperation and affect patient prognosis [36]. There is no study that clearly explains the reason for the occurrence of EFC. However, Kim et al. reported that epidural fluid collection occurs due to CSF leakage through a dura defect or accumulation of exudate from the temporalis muscle and myocutaneous tissue injury during dissection [37]. EFC risk factors have been reported as postoperative epidural air bubble, dural calcification, and size of the skull defect [37].

In our study, postoperative epidural air bubbles had the same risk factor (OR 3.809, 95% CI 1.383–10.490, *p* = 0.010) as in previous studies, and was 0.294-times lower in the presence of the silicone elastomer sheet (OR 0.294, 95% CI 0.093–0.934, *p* = 0.039). Postoperative epidural air bubble is related to the skull bone defect size. In our study, postoperative epidural air bubble was thought to be more likely because it included large-sized bone flaps with an anterior-posterior diameter of 12 cm or more. In addition, when silicone elastomer sheets were used, since dura repair was performed at a similar frequency between the two groups, it is thought that EFC decreased by reducing exudate from the temporalis muscle and myocutaneous tissue injury rather than EFC caused by dura injury.

In trauma patients, the frequency of complications may differ due to invisible dura injury and muscle injury. Matt Pierson et al. reported that there was no statistical difference in the time of cranioplasty, operative time, blood loss, length of stay, and complications when comparing 37 trauma and cerebrovascular patients who used ePTFE as an anti-adhesion material [20]. In our study, there was no statistical difference between the trauma and non-trauma groups. Therefore, the use of the sheet was not limited. However, as the number of patients in the comparative control group was small, additional studies are needed.

### 4.4. Cost-Effective Silicone Elastomer Sheet

The silicone elastomer sheet is relatively inexpensive and easily operable. At our institution, the cost for the sheet is approximately USD 50, which is cheaper than other anti-adhesive materials and more cost-effective [15,28,38]. Although not statistically significant, the mean hospital follow-up period in group B (mean = 57 days) was shorter than that in group A (mean = 75 days). Additional research on the specific cost of examination and the number of outpatient treatments is needed. Nevertheless, we believe that the use of silicone elastomer sheets will improve patient convenience and reduce medical costs.

### 4.5. Limitations of the Study

This study had a few limitations. First, the study was a retrospective review conducted on a relatively small number of patients at a single institution. Retrospective studies are associated with an inherent bias. When comparing trauma and non-trauma patients, the scale was small, so a larger scale study is needed. Second, the results from the study cannot be compared with other anti-adhesive materials. Large, prospective, randomized, multicenter trials are required. Third, although the baseline characteristics of diabetes mellitus and anticoagulant/antiplatelet agent medication factors were not statistically significant, they are factors that may have affected the results. In the case of CP surgery, we believe that the baseline characteristics of diabetes mellitus and anticoagulant/antiplatelet agent medications would not have a significant effect because CP proceeded as regular surgery to adjust the sugar level and restrict the effect of anticoagulant/antiplatelet agent medication. In particular, the use of anticoagulant/antiplatelet agents can affect EBL, so all of the patients stopped taking anticoagulant/antiplatelet medications a week before performing CP surgery. However, this point can be a weak point, so further research is needed. Fourth, the long follow-up failed to reflect major complications such as bone resorption. Nevertheless, this study had some remarkable findings and suggestions.

## 5. Conclusions

The technique using the silicone elastomer sheet allows surgeons to easily dissect the surgical plane during CP, which shortens the operation time, reduces EBL, and minimizes complications of EFC. Moreover, there was no infection and no adhesion even after delayed surgery. Although additional research may be required, the use of a cost-effective silicone elastomer sheet is a good and safe option.

## Figures and Tables

**Table 1:** The baseline characteristics of patients in the two groups.

	Group A (without Silicone Elastomer Sheet) (*n* = 51)	Group B (with Silicone Elastomer Sheet) (*n* = 30)	*p*-Value
Age at the time of cranioplasty (years)	56.3 ± 13.1	57.0 ± 13.1	0.848
Sex			0.877
Male	28 (54.9)	17 (56.7)	
Female	23 (45.1)	13 (43.4)	
Underlying disease			
DM	12 (23.5)	2 (6.7)	0.053
HTN	20 (39.2)	11 (33.3)	0.820
Liver Disease	2 (3.9)	1 (3.3)	0.892
Kidney Disease	3 (5.9)	0 (0)	0.176
Trauma/Non-trauma			0.430
Trauma	16 (31.4)	12 (40.0)	
Non-trauma	35 (68.6)	18 (60.0)	
Anticoagulant/antiplatelet agent medication	12 (23.5)	2 (6.7)	0.053

M: Male, F: Female, DM: Diabetes mellitus, HTN: Hypertension.

**Table 2:** Difference between complications and midline shift between the two groups after DC.

	Group A (without Silicone Elastomer Sheet) (*n* = 51)	Group B (with Silicone Elastomer Sheet) (*n* = 30)	*p*-Value
Anticoagulant/antiplatelet agent medication	12 (23.5)	2 (6.7)	0.053
EDH/SDH after DC	5 (9.8)	4 (13.4)	0.551
Revision surgery for EDH/SDH after DC	4 (7.8)	1 (3.3)	0.415
Midline Shift Difference (1 week-immediate postoperative) (mm)	3.8 ± 3.1	3.2 ± 4.6	0.423

EDH: Epidural hemorrhage, SDH: Subdural hemorrhage, DC: Decompressive craniectomy.

**Table 3:** CP results in the two groups.

	Group A (without Silicone Elastomer Sheet) (*n* = 51)	Group B (with Silicone Elastomer Sheet) (*n* = 30)	*p*-Value
Timing for CP (days)	85.0 ± 48.4	72.8 ± 40.8	0.251
Division for early/late CP (days)			0.260
Early surgery (<90)	33 (64.7)	23 (76.7)	
Late surgery (≥90)	18 (35.3)	7 (23.3)	
Transfusion	2 (3.9)	0	0.272
Hb difference (Postoperative-Preoperative) (mg/dL)	1.0 ± 0.7	0.9 ± 0.6	0.705
Estimated blood loss (mL)	235.2 ± 189.8	133.3 ± 99.4	0.002
Operation time (minutes)	129.1 ± 35.9	98.3 ± 37.8	<0.001
Epidural fluid collection	21(41.2)	5 (16.7)	0.023
With midline shift	4(7.8)	1 (3.3)	0.415
Epidural fluid collection depth (mm)	8.7 ± 3.7	9.6 ± 4.3	0.637
Infection	2 (3.9)	2 (6.7)	0.581
Brain cortical injury	3 (5.9)	1 (3.3)	0.609
Hydrocephalus	2 (3.9)	0	0.272
Revision operation for Complication	3 (5.9)	2(6.7)	0.736
Hospital admission periods (day)	13.0 ± 3.6	12.4 ± 4.3	0.555
Follow up periods (day)	75.6 ± 72.2	57.8 ± 72.6	0.286
Postoperative epidural air bubble	22 (43.1)	10 (33.3)	0.383
Dural Calcification	8 (15.6)	2 (6.6)	0.233

CP: Cranioplasty.

**Table 4:** Result of epidural fluid collection after cranioplasty.

	EFC (+) (*n* = 26)	EFC (−) (*n* = 55)	*p*-Value
Sex			0.790
Male	15 (57.7)	30 (54.5)	
Female	11 (42.3)	25 (45.5)	
Age	58.1 ± 10.9	55.9 ± 14.7	0.500
Trauma	10 (38.5)	18 (32.5)	0.612
Non-trauma	16 (61.5)	37 (67.3)	
Silicone elastomer sheet presence	5 (19.2)	25 (45.5)	0.023
Operative time (min)	118.4 ± 35.0	107.8 ± 38.8	0.239
Epidural air bubble after CP	16 (61.5)	16 (29.1)	0.005
Dural calcification	6 (23.1)	4 (6.8)	0.044
CP timing			0.041
Early CP	14 (53.8)	42 (76.4)	
Late CP	12 (46.2)	13 (23.7)	
Intraoperative dura repair	13 (50.0)	37 (67.3)	0.135

EFC: Epidural fluid collection, CP: Cranioplasty.

**Table 5:** Multivariate regression analysis for risk factors of epidural fluid collection.

	OR	95% CI	*p*-Value
Silicone elastomer sheet presence	0.294	0.093–0.934	0.038
Epidural air bubble after CP	3.809	1.383–10.490	0.010

OR: Odds ratio, CI: Confidence interval.

**Table 6:** Comparison of results in trauma and non-trauma in Group B.

	Trauma (*n* = 12)	Non-Trauma (*n* = 18)	*p*-Value
Estimated blood loss (mL)	158.3 ± 122.1	108.3 ± 64.7	0.260
Operation time (minutes)	105.8 ± 45.8	93.3 ± 31.8	0.384
Complication: Epidural fluid collection	2 (16.5)	3 (16.7)	0.387
Complication: Infection	1 (8.3)	1 (5.6)	0.497
MLS difference after DC	3.0 ± 3.0	3.0 ± 5.6	0.995

MLS: Midline shift, DC: Decompressive craniectomy.

## Data Availability

Data is available on request.
